# A Review of COVID-19 in Relation to Metabolic Syndrome: Obesity, Hypertension, Diabetes, and Dyslipidemia

**DOI:** 10.7759/cureus.27438

**Published:** 2022-07-29

**Authors:** Elias Makhoul, Joseph L Aklinski, Jesse Miller, Cara Leonard, Sean Backer, Payal Kahar, Mayur S Parmar, Deepesh Khanna

**Affiliations:** 1 Foundational Sciences, Nova Southeastern University Dr. Kiran C. Patel College of Osteopathic Medicine, Clearwater, USA; 2 Osteopathic Medicine, Nova Southeastern University Dr. Kiran C. Patel College of Osteopathic Medicine, Tampa, USA; 3 Health Sciences, Florida Gulf Coast University, Fort Myers, USA

**Keywords:** overweight, obesity paradox, ace-2 receptor, sars-cov-2, dyslipidemia, diabetes, hypertension, obesity, covid-19, metabolic syndrome (mets)

## Abstract

Although severe cases and mortality of coronavirus disease 2019 (COVID-19) are proportionally infrequent, these cases are strongly linked to patients with conditions of metabolic syndrome (obesity, hypertension, diabetes, and dyslipidemia). However, the pathophysiology of COVID-19 in relation to metabolic syndrome is not well understood. Thus, the goal of this secondary literature review was to examine the relationship between severe acute respiratory syndrome (SARS-CoV-2) infection and the individual conditions of metabolic syndrome.

The objective of this secondary literature review was achieved by examining primary studies, case studies, and other secondary studies, to obtain a comprehensive perspective of theories and observations of COVID-19 etiology with metabolic syndrome. The most extensive research was available on the topics of diabetes, hypertension, and obesity, which yielded multiple (and sometimes conflicting) hypothetical pathophysiology. The sources on dyslipidemia and COVID-19 were scarcer and failed to provide an equally comprehensive image, highlighting the need for further research.

It was concluded that hypertension had the strongest correlation with COVID-19 incidence (followed by obesity), yet the causative pathophysiology was ambiguous; most likely related to cardiovascular, angiotensin-converting enzyme 2 (ACE-2)-related complications from renin-angiotensin-aldosterone system (RAAS) imbalance. Obesity was also positively correlated to the severity of COVID-19 cases and was believed to contribute to mechanical difficulties with respiration, in addition to hypothetical connections with the expression of ACE-2 on abundant adipose tissue. Diabetes was believed to contribute to COVID-19 severity by producing a chronic inflammatory state and interfering with neutrophil and T-cell function. Furthermore, there were indications that COVID-19 may induce acute-onset diabetes and diabetic ketoacidosis. Lastly, dyslipidemia was concluded to potentially facilitate SARS-CoV-2 infection by enhancing lipid rafts and immunosuppressive functions. There were also indications that cholesterol levels may have prognostic indications and that statins may have therapeutic benefits.

## Introduction and background

An initial case of pneumonia with unknown etiology was seen in December 2019, in Wuhan, China [1]. The cause of the infection was later identified as a novel coronavirus, named severe acute respiratory syndrome coronavirus 2 (SARS-CoV-2) (due to its genetic similarity to the severe acute respiratory syndrome (SARS) virus), the pathogen of the disease COVID-19 [2]. Since the initial case in Wuhan, SARS-CoV-2 has propagated a global pandemic with approximately 527,857,044 reported cases of COVID-19 worldwide, resulting in nearly 6,301,101 casualties. In the United States alone, there have been over 85,014,373 confirmed cases, with roughly 1,028,946 deaths (as of May 23, 2022) [3].

SARS-CoV-2 is thought to have initially undergone zoonotic transmission (animal-to-human) via an infected bat linked to a wet animal market [4]. Coronaviruses often affect humans and animals, causing illnesses such as the common cold. However, their past coronavirus outbreaks have also resulted in more severe symptomology, such as those of severe acute respiratory syndrome (SARS) and Middle Eastern respiratory syndrome (MERS) [5,6].

SARS was first identified in February of 2003 in the Guangdong province in China. Similar to SARS-CoV-2, the SARS coronavirus was likely also zoonotically transmitted from either an infected bat or civet cat [5]. MERS-CoV was first identified in Saudi Arabia in September of 2012 and was proposed as initially transmitted to humans from camels [6]. Both conditions generally caused mild symptoms such as cough, shortness of breath, diarrhea, and fever, but could in severe cases lead to respiratory distress, and death [5,6]. The mortality rate for SARS is approximately 15% [7], and 35% for MERS [6].

Aside from initial zoonotic transmission, SARS-CoV-2 is primarily transmitted person-to-person through respiratory droplets of infected individuals. Respiratory droplets can be transmitted when a person speaks, coughs, or sneezes, and may land directly on a recipient’s integument or mucosal surfaces, be inhaled, or contaminate objects (fomites). SARS-CoV-2 infects individuals by attaching to the angiotensin-converting enzyme 2 (ACE2) receptor [8,9], which can be found in lung epithelial cells [4]. SARS-CoV-2 appears to be more contagious than influenza and has been demonstrated to be transmitted by individuals with asymptomatic presentation highlighting the necessity for individuals to limit close contact with others [10]. The reproductive ratio (R0) of SARS-CoV-2 is estimated with a mean and median of 3.28 and 2.79, respectively (range from 1.5 to 6.68) [11].

Symptoms of the disease usually appear 2-14 days after initial exposure, with an average onset of five days [12-14]. Like other coronaviruses, SARS-CoV-2 generally causes unspecific symptoms of fever, muscle pain, shortness of breath, cough, headache, loss of smell, fatigue, and diarrhea. In some cases, it may progress to more severe symptoms, such as trouble breathing, chest pain or pressure, and confusion [15]. Critical status and mortality of COVID-19 are usually due to acute respiratory distress syndrome (ARDS), septic-organ failure, respiratory insufficiency, secondary to initial viral pneumonia [16]. A hypercoagulable state, with microthrombi in organ vasculature and pulmonary thromboembolism, has also been frequently observed in severe COVID-19 cases, yet the relationship to mortality remains unclear [16]. Initial studies of COVID-19 highlight respiratory failure as the predominant cause of morbidity and mortality. However, there is increasing evidence to support the importance of comorbidities as key factors of morbidity and mortality [17].

COVID-19 has effects on different sub-populations such as obese, pregnant women and newborns, etc [18,19]. Elevated risk of severe presentations of COVID-19 has been strongly associated with patients with cardiovascular disease, diabetes, hypertension, and who are overweight or obese [20,21]. Metabolic syndrome is a combination of conditions occurring simultaneously, increasing the risk for future cardiovascular disease. The conditions include elevated blood pressure, elevated blood glucose, excess waist circumference, subcutaneous abdominal fat, and abnormal cholesterol or triglyceride levels. It is estimated that 23% of adults in the United States suffer from metabolic syndrome [22]. Metabolic syndrome may have a significant effect on the pathophysiology of COVID-19. Hypertension and diabetes have both been linked to altered expression of the ACE2 receptor, leading to increased affinity and susceptibility for SARC-CoV-2 [23-26]. Additionally, diabetes can lead to a chronic inflammatory state, resulting in tissue inflammation and ineffective activation of immune responses, and killing of pathogens [26-28]. Overweight and obesity may be risk factors for COVID-19, as increased waist circumference is associated with reduced lung and/or chest wall compliance, potentially compounding the consequences of respiratory viruses such as SARS-CoV-2 [29]. Additionally, the ACE2 receptor is expressed in adipose tissue, which is found in abundance in overweight and obese individuals. This increased level of ACE2 receptor may result in increased attachment points for SARS-CoV-2 to infect the host [30,31]. Overweight or obese individuals also have reduced nitric oxide (NO) and other anti-inflammatory mechanisms, which may increase susceptibility to pathology by SARS-CoV-2 [32]. With an increase in adipose tissue, there is a chronic state of low-grade inflammation, which also impairs the immune system [33]. Lastly, hyperlipidemia may be a risk factor for COVID-19, as cholesterol may facilitate the binding of SARS-CoV-2 to ACE2 receptors through lipid rafts [34]. This review will further examine the relationship between metabolic syndrome and pathophysiology and the clinical severity of COVID-19.

## Review

Methods

A narrative literature review of COVID-19 in relation to metabolic syndrome was conducted. The narrative literature review encompassed four separate investigations of COVID-19 in relation to obesity, hypertension, diabetes, and dyslipidemia, which were all included as separate sections in this study. The literature review was conducted by using online databases such as PubMed, MEDLINE, Google Scholar, and Web of Science.

During the literature search for COVID-19 and obesity, the following search terms were utilized: metabolic syndrome, obesity, COVID-19, and BMI. Inclusion criteria encompassed primary studies that discussed incidence/prevalence, pathophysiology, complications, or management of obesity as comorbidity or the obesity paradox in COVID-19. Both outpatient and inpatient studies and all geographic locations were included. Exclusion criteria included pediatric populations, non-English language text, and studies with no subject classification based on BMI. Based on the above criteria, 14 primary articles were included in this study.

In the literature search for COVID-19 and hypertension, the following search terms were used: metabolic syndrome, hypertension, COVID-19, and blood pressure. Inclusion criteria were primary research of hypertension incidence/prevalence, complications, pathophysiology, or management/medication as COVID-19 comorbidity. Studies were included with subjects grouped based on blood pressure and with clear measurements of COVID-19 outcomes. Both inpatient and outpatient were included. Studies in other languages were excluded, but all geographic regions were included. Pediatric cohorts were excluded, as well as studies with no clear, consistent, and replicable measurements of the blood pressure of subjects. Based on the above criteria, 11 primary studies were included.

The literature search for diabetes and COVID-19 was based on the following search terms: metabolic syndrome, diabetes, diabetes mellitus, COVID-19, and insulin resistance. Primary research was included if it discussed incidence/prevalence, pathophysiology, complications, management/medications, or diabetogenic effect of diabetes as a comorbidity. Inclusion criteria were primary research, with a clear, consistent, and replicable classification of pre-existing diabetes mellitus among subjects with concurrent COVID-19 infection. Pediatric cohorts, diabetes insipidus, and studies in other languages were excluded. All geographic regions were included. Ultimately, the above criteria yielded 10 primary articles that were included.

The literature review for dyslipidemia was based on the following search terms: COVID-19, dyslipidemia, hyperlipidemia, cholesterol, and triglycerides. Primary research was included that discussed incidence/prevalence, pathophysiology, complications, or management/medications of dyslipidemia as a comorbidity of COVID-19. Only studies describing numerical measurements of serum lipid levels in COVID-19 patients were included. Pediatric populations were excluded, non-English text was excluded, and studies with no statistical analysis of serum lipid levels in relation to the aforementioned COVID-19 variables (i.e. incidence, prevalence, outcomes, etc.) were excluded. Ultimately, eight primary studies were included based on the above criteria.

Comprehensive statistical data related to COVID-19 cases and deaths were obtained from the Centers for Disease Control and Prevention (CDC) and the World Health Organization (WHO) websites. All searches were performed by all the investigators mentioned and re-checked for accuracy on two different occasions. The searches have been restricted to publications from 2010 to 2022

COVID-19 and obesity

Incidence and Prevalence

Obesity is clinically defined as having a BMI of 30 kg/m^2^ or above [35]. It is considered a contemporary health epidemic and is a known risk factor for a variety of cardiovascular and respiratory diseases [29,36]. The link between COVID-19 and obesity has been documented throughout the pandemic [30-32]. The WHO reports that approximately 650 million people over the age of 18 are considered obese worldwide as of 2016 [37]. Age-adjusted obesity prevalence in the United States is 42%, according to data from the National Health and Nutrition Examination Survey (NHANES) [38-40]. This may be one factor explaining the high rate of COVID-19 infection in the United States population [41]. The Coronavirus Disease-Associated Network Surveillance Tool (COVID-NET) was a population surveillance tool used by the CDC to gather information about COVID-19 hospitalization rates across various states of the United States in March. Obesity represented 48.3% of the underlying conditions among those admitted [38]. Obesity was second only to hypertension among underlying conditions in the previous data. For individuals in the 18-49 and 50-64 age groups, obesity was the most common underlying condition [38].

In a New York-based cohort study of 22,254 subjects, overweight and obese individuals were found to be significantly more likely to test positive, become hospitalized, and suffered from an elevated mortality rate (albeit not significant) [42]. In contrast, a United Kingdom (UK) cohort study of 20,133 hospitalized patients found a statistically significant elevated mortality risk (hazard ratio of 1.33 by multivariable Cox proportional hazards model) among obese patients [43]. However, the UK study did note a decreased prevalence of obesity in the cohort (11%) compared to the general UK population (29%) [43]. The relationship between COVID-19 and obesity varies somewhat between different studies. Perhaps, obesity is correlated with poor COVID-19 outcomes mainly due to its association with other high-risk comorbidities such as hypertension, cardiovascular disease, and diabetes mellitus. Nevertheless, a large meta-study, by Yang et. al. (2020), summarized clinical outcomes of nearly 164,622 subjects from numerous studies from various global regions and found obesity increased the incidence of positive COVID-19 test results, hospitalization, ICU admission, invasive mechanical ventilation, and in-hospital mortality [44]. Furthermore, the degree of obesity (determined by BMI categories) was positively correlated with an increased incidence of all the aforementioned outcomes [44]. The Yang et. al. review indicates a clear global correlation between increasing BMI and poor clinical outcomes, regardless of exact pathophysiology and secondary comorbidities.

Pathophysiology

Obesity has been clinically correlated with worse outcomes of many viral infections and was associated with higher rates of mortality in the 2009 H1N1 outbreak [32]. Central obesity, which refers to a fat accumulation primarily in the abdomen and thorax, has been proven to impair respiratory function by reducing lung and/or chest wall compliance [29]. It is therefore not surprising that respiratory viruses such as SARS-CoV-2, the cause of COVID-19, may have a greater impact on individuals with this fat distribution pattern, which is specific to the metabolic syndrome.

It has been well-documented that the ACE2 receptor is the primary method by which COVID-19 invades lung epithelial cells [4]. ACE2 is also present on adipose tissue, which studies have suggested may have higher expression of ACE2 than lung tissue. This may be another mechanism explaining the increased susceptibility of COVID-19 in obese individuals, as more adipocytes are present [30,31]. However, this mechanism of pathophysiology is somewhat questionable, as SARS-CoV-2 has been observed to predominantly infect cells of the lower respiratory tract [4,45]. Additionally, ACE2 is present on endothelial cells, which has drawn speculation that those with vascular abnormalities are at a greater risk of COVID-19 [32]. Hyperinsulinemia, insulin resistance, as well as increased circulating leptin in obesity may all act to reduce NO (via reduced expression of nitric oxide synthase) and other cytokines that have anti-inflammatory action. This might make the endothelium more susceptible to invasion and disruption by SARS-CoV-2 [32]. 

Aside from ACE2 expression, a chronic state of low-grade inflammation is also induced by increased adipose tissue, which may impair immunity [33]. A diet high in saturated fats may precipitate this hyperinflammatory state. In the United States as well as many European countries, a diet high in saturated fatty acids are typically consumed and has been termed the Western Diet. This diet has been shown to simultaneously activate the innate immune system while suppressing adaptive immunity [46]. In a study, mice that were fed a diet high in saturated fats were shown to have exacerbated airway inflammation including an increased number of macrophages infiltrating the lung alveoli, via an innate immune response [47]. This may have implications for COVID-19, as excessive alveolar damage is an indicator of severe disease and usually precedes more aggressive treatments. T and B lymphocytes, which are the principal cells in adaptive immunity, also have reduced function in individuals consuming the Western diet [46]. Adaptive immunity is necessary to fight against viral pathogens, so impairment may render an individual more vulnerable to COVID-19 infection. These mechanisms of obesity altering immune system function have been implicated in previous outbreaks of influenza [48].

An excessive host immune reaction to SARS-CoV-2 may trigger severe complications such as ARDS. MicroRNAs, which are non-coding RNAs that suppress target gene expression, have been shown to play a significant role in infection by respiratory viruses including human coronaviruses. In particular, miR-146a is active early during viral infection and may limit excessive inflammatory responses by regulating toll-like receptor downstream signaling. Downregulation of circulating miR-146a was found in patients with obesity, as well as patients with diabetes and hypertension. This has led to the hypothesis that this deficiency in miR-146a may be one possible contributing factor to the higher rates of mortality, severe progression, and hospitalization in obese patients [49].

Precise mechanisms of how the hyperinflammatory state seen in obesity may lead to a more severe case of COVID-19 is still under investigation. Additionally, further research is being conducted to determine how certain therapeutics may limit excessive inflammation in obese patients to reduce the potential for complications [32].

Complications

Obesity has been linked to a worse prognosis of COVID-19 infection, including an increased risk for complications and death [49-56]. Cai et al. followed 383 hospitalized COVID-19 patients in a hospital in Shenzhen, China, separating patients into groups based on the severity of the disease. Severe COVID-19 was defined by either respiration ≥ 30 per min, O2 saturation ≤ 93%, partial pressure of oxygen/fraction of inspired oxygen (P/F ratio) ≤ 300 mmHg, or organ failure requiring ICU monitoring [51]. The results of this study revealed that obese patients were 3.40 times more likely to progress to severe COVID-19 [51].

The primary complication associated with COVID-19 is hypoxic respiratory failure, which may require endotracheal intubation and invasive mechanical ventilation [52]. The need for mechanical ventilation is used as a marker for progression to severe disease in many studies. A Chicago study of 486 COVID-19 patients found that 28.1% required intubation. Of those intubated, age and BMI were the only factors associated with increased time to extubation [52]. A similar study of 124 intensive care patients admitted to a French hospital found that invasive mechanical ventilation was required most frequently in those with BMI>35, with nearly 90% of these patients eventually requiring intubation [53]. Obesity has also been linked to increased mortality from COVID-19, especially when present with other underlying illnesses [54]. An early study conducted in Wuhan, China, that followed 112 COVID-19 patients with cardiovascular disease (CVD) revealed that the average BMI of patients receiving critical care was significantly higher than the patients who had a more standard hospital course [54]. In terms of mortality, 88% of COVID-19 patients with CVD that did not survive had a BMI>25, as opposed to only 19% with BMI>25 who did survive [54].

Age has been an important factor in considering the risk of severe progression and death from COVID-19. Several studies have suggested that obesity may be just as important as age to predict hospitalization, more severe progressions, and higher mortality rates. Among a group of patients admitted to a special COVID-19 ward in an Italian hospital, those that were overweight/obese were on average younger than those admitted who were normal weight [57]. In a retrospective study conducted in New York City (NYC), United States, of 3,615 patients who tested positive for COVID-19, 21% had a BMI of 30-34, and 16% with a BMI >35 [50]. This NYC study concluded that obesity was a risk factor for hospitalization in patients under 60 years of age [50]. Patients under 60 with a BMI>30 were multiple times more likely to require admission when compared to COVID-19 patients in the same age group with BMI<30 [50]. A similar NYC study of 5,279 patients who tested positive revealed that patients with a BMI>40 were several times more likely to be hospitalized and more likely to develop critical diseases requiring more advanced treatment [58]. 

In a study focused on the association between BMI and the rates of adverse outcomes in 25,952 COVID-19 patients at United States Veterans Affairs (US VA) hospitals, class 3 obesity (the highest class of obesity, generally defined as having a BMI >40) was associated with a higher risk of mechanical ventilation and mortality. Further, these associations were present primarily in patients under 65 years of age, an age group generally thought to be of comparable lower risk, and were attenuated or absent in older age groups [55]. Similarly, obesity (along with diabetes with chronic complications and hypertension with chronic complications) was associated with higher mortality risk, particularly among 20-39-year-olds, in a large study on 66,646 hospital admissions with a COVID-19 diagnosis across 613 United States hospitals [56].

Management

Obesity intervention and prevention are centered around behavioral changes to improve diet and increase physical activity.

The Mediterranean diet is widely considered a standard for the prevention of obesity and associated complications of cardiovascular disease and insulin resistance. The abundance of polyphenols (particularly flavonoids) in the diet has anti-inflammatory, anti-oxidant, and anti-thrombotic properties that can be highly protective against exaggerated inflammatory and procoagulant states of severe COVID-19. Much of these effects are mediated via inhibition of nucleic factor kappa-beta (NF-kB) and inhibition of proinflammatory cytokines such as interleukin-6 (IL-6) and tumor necrosis factor-alpha (TNF-a) [59].

Despite a lack of extensive research on the relationship between exercise and COVID-19 pathophysiology, there are indications that moderate levels of physical exercise decrease the incidence of acute respiratory illness, lower duration, and symptom severity, and decrease mortality from COVID-19 [45,60]. A moderate exercise level is generally considered as 150-300 minutes of aerobic exercise (65-75% maximum heart rate) and two resistance training sessions per week [60]. Some of these effects could stem from the immune system response due to exercise. During exercise, increased cardiac output improves circulation and tissue exchange of immune cells, immunoglobulins, and cytokines, thus promoting active immune surveillance and response. In addition, dynamic exercise increases cardiac output and catecholamine activity, consequently mobilizing effector lymphocytes to aid in immune responses [45]. Regularly, physical exercise has been shown to modulate/decrease systemic inflammatory responses and stress hormones, while simultaneously increasing levels of lymphocytes, immature B-cells, NK cells, and monocytes. The result may promote immune vigilance yet prevent risks of excessive systemic immune responses such as those seen in ARDS [45].

In severe cases of obesity (BMI over 40 kg/m^2^), bariatric surgery (or other surgical intervention) may be indicated as a form of management. Research indicates that a history of previous bariatric surgery in an obese (BMI > 30 kg/m^2^) COVID-19 patient is independently associated with decreased mortality and incidence of mechanical intubation [61].

Obesity Paradox

It is interesting to note the existence of a phenomenon referred to as the “Obesity Paradox”, referring to a lower mortality rate in patients with obesity with certain conditions in several studies. In one study, the mortality rate of obese patients with ARDS and other acute respiratory conditions was lower when compared to patients with non-obese BMIs [62]. One hypothesis for this occurrence is that the chronic state of inflammation seen in obese individuals (particularly of class I obesity) may provide a protective measure [63]. However, this phenomenon is not widely documented among COVID-19 patients, despite the potential severe progression of COVID-19 to ARDS. Several reasons have been postulated, one being those obese individuals who may normally be considered high risk (thus treated earlier and more aggressively) may no longer have access to early treatment due to the shortage of hospital beds and clinicians amidst the COVID-19 pandemic [64,65]. Ultimately, the uncertainty regarding “obesity paradox” in COVID-19 stems from a lack of adequate research. Further research on the obesity paradox in COVID-19 could provide more clarity on the prioritization of patient risk groups. Nevertheless, the current foundation of research generally demonstrates obesity as a predictive factor for the severity of morbidity and mortality. The research highlights the necessity of obesity prevention to reduce the risk of poor COVID-19 clinical outcomes. In addition to practicing proper hygiene and adhering to social distancing guidelines, maintaining a regular exercise regimen and a healthy diet should be considered important preventative measures. Table 1 gives a summary of cohort studies of COVID-19 patients in relation to BMI and obesity. Table 1 shows the compilation of studies of COVID-19 patients in relation to BMI and Obesity, in China, Italy, France, the United Kingdom, and the United States.

**Table 1:** Summary of cohort studies of COVID-19 patients in relation to BMI and obesity, in China, Italy, France, the United Kingdom, and the United States NYC: New York City; COVID-19: coronavirus disease 2019;  CVD: cardiovascular disease; VA: Veterans Affairs

Study	Patient Population	General Findings
Marcello et al., 2020 [42]	13,442 COVID-19 positive individuals (of 22,254 tested). 6,248 individuals were hospitalized, of which 1724 deceased. Patients were part of the NYC health and hospital public health system.	Overweight and obese individuals were significantly more likely to test positive and to be hospitalized. Obese hospitalized patients had a higher (but not significant) mortality rate than normal-weight patients (11% vs 8%).
Docherty et al., 2020 [43]	20,133 COVID-19 positive in-patients throughout the United Kingdom.	Obese (BMI > 30 kg/m^2^) in-patients had a statistically significant elevated mortality hazard ratio of 1.33, compared to normal-weight patients.
Busetto et al., 2020 [57]	92 patients in COVID-19 ward of an Italian hospital.	Overweight and obese patients were, on average, younger than normal-weight patients. Despite younger age, obese patients required more frequently assisted ventilation
Lighter et al., 2020 [50]	3,615 COVID-19 positive individuals under 60 who presented to a large academic hospital system in NYC.	775 with BMI of 30-34, and 595 with BMI>35. Obese patients under 60 were more likely to be admitted to acute and critical care.
Petrilli et al., 2020 [58]	5,279 patients hospitalized with COVID-19 in NYC	Obesity (BMI>30) was associated with an increased risk of hospital admission. The strongest risk factors predicting critical illness were age, heart failure, male sex, and BMI>40.
Cai et al., 2020 [51]	383 consecutively admitted COVID-19 patients in a hospital in Shenzhen, China	Obese patients were 3.40 times more likely to progress to severe Covid-19 requiring ICU admission
Hur et al., 2020 [52]	486 COVID-19-positive patients hospitalized across 10 hospitals in Chicago	Median BMI was 30.6. 138 total intubated patients with 78 extubated. BMI associated with increased time to extubation.
Simonnet et al., 2020 [53]	124 consecutively admitted ICU patients with COVID-19 in a French hospital	Obesity (BMI>30) seen in 75% of ICU patients. Need for invasive mechanical ventilation (IMV) significantly associated with BMI, independent of age, diabetes, and hypertension.
Peng et al., 2020 [54]	112 COVID-19 positive patients with underlying CVD in Wuhan, China	BMI of patients requiring critical care was significantly higher than non-critical care group. In addition, 88% of patients who did not survive had a BMI>25, as opposed to 19% with BMI>25 who did survive.
Eastment et al., 2020 [55]	25,952 SARS-CoV-2 positive patients from VA hospitals across the United States	Patients with a higher BMI were more likely to test positive for SARS-CoV-2. They were also more likely to need mechanical ventilation, as well as statistically more likely to die from infection. This correlation was seen in patients under 65 years of age and were attenuated or absent in patients over 65 years of age.
Goodman et al,, 2020 [56]	66,646 COVID-19 inpatients across 613 United States hospitals	Obesity, diabetes with chronic complications, and hypertension with chronic complications were risk factors in most age-groups with the highest relative risks among 20-39 year old. Male sex was also independently associated with higher mortality risk.
Gao et al., 2020 [18]	150 adult COVID-19 positive inpatients from three Chinese hospitals	The cohort was divided into 75 obese patients (BMI > 25) and 75 non-obese patients. In the obese cohort, 33.3% of patients developed severe COVID-19 pathology, compared to 14.7% of patients in the non-obese cohort. The findings were statistically significant. Median duration of hospitalization was also increased in the obese cohort.
Kass et al., 2020 [66]	A retrospective cohort of 265 COVID-19 patients admitted to the ICU at six United States academic hospital systems	There was a significant negative correlation between BMI and age among ICU admitted COVID-19 patients. Younger patients, on average, had a higher BMI than older patients. Only 25% of the COVID-19 patients admitted to the ICU had a BMI < 26.
Klang et al., 2020 [67]	3,406 COVID-19 patients admitted to a large academic hospital system in New York, United States	Cohort was subdivided into a group of patients younger than 50 years old, and one of older than 50. There was a stronger positive correlation between obesity (BMI > 40) and mortality among patients under 50, than for patients older than 50. Essentially, obesity was a strong risk factor for mortality, particularly in younger patients.

COVID 19 and hypertension

Incidence and Prevalence

As the COVID-19 pandemic has progressed, more studies have examined the correlation between incidence/prevalence of infection and various comorbidities, such as hypertension and cardiovascular disease [4]. A common trend has emerged of hypertension being the most prevalent comorbidity found throughout multiple studies [4,14,15,68]. More specifically, one recent meta-analysis study found hypertension was the most prevalent COVID-19 comorbidity among non-hospitalized patients (32%), intensive care patients (26%), and fatalities (35%) [4]. Another meta-analysis of 15,302 COVID-19 patients found a 38.2% prevalence of hypertension [68], compared to an estimated 31.1% prevalence in the general population globally [69]. One of the earliest and most extensive studies on hypertension and COVID-19, on a New York-based cohort of 5700, stated hypertension as the most common comorbidity at 56.6% of hospitalized patients [70]. Even more astounding, a New Orleans-based study of 800 hospitalized patients found a 73.8% prevalence of hypertension [71]. An Argentinian database analysis of 207,079 positive polymerase chain reaction (PCR)-tested individuals, found an overall hypertension prevalence of 19.2% and 33.9% among hospitalized patients: the highest among analyzed comorbidities [72]. Although the prevalence varies between studies, they are likely due to differences in the definition of hypertension (stage 1 or stage 2), cohort sampling (non-hospitalized or hospitalized), and geographical differences in the prevalence of hypertension. Table 2 shows the compilation of cohort studies of COVID-19 patients in relation to hypertension.

**Table 2:** Summary of studies of COVID-19 patients in relation to hypertension MERS: Middle East respiratory syndrome; COVID-19: coronavirus disease 2019

Study	Sample Size (N)	Hypertension (N, %)	Significant findings related to hypertension
Schönfeld et al., 2021 [72]	207,079	39,833 (19.2)	Prevalence of hypertension increased with severity of COVID-19 illness. 54.2% of deceased patients suffered from hypertension.
Yoshida et al., 2021 [71]	776	573 (73.8)	Comorbidities had a more significant effect on clinical outcome in women compared to men.
Richardson et al., 2020 [70]	5,700	3026 (56.6)	Hypertension was associated with a higher mortality rate than the cohort overall.
Li et al. 2020 [73]	1,527	261 (17)	Statistically significant higher case rate in ICU patients with hypertension.
Espinosa et al. 2020 [74]	16,222	12,319 (32)	Comorbidities increase death rate probability by 2.4 times
Rodriguez-Morales et al., 2020 [75]	656	122 (18.6)	The most prevalent comorbidity was hypertension
Yang et al., 2020 [25]	1,576	333 (21.1)	Increased risk of death in a variety of respiratory infections including, COVID-19, influenza, and MERS
Grasselli et al. 2020 [76]	1,591	509 (49)	Hypertension was most common comorbidity and correlated with higher mortality
Huang et al., 2020 [77]	310	113 (36.5)	Patients with hypertension had higher mortality, higher proportion of non-invasive mechanical ventilation, severe cases, and ICU admissions.
Barrera et al. 2020 [78]	15,794	2,685 (17)	The prevalence of hypertension as a comorbidity was 17% in all hospitalized patients, and 32% in severe COVID-19 cases
Katz 2020 [79]	3,222	519 (16.1)	Young adults (18-34 years) with hypertension faced similar risk of severe COVID-19 disease pathology as middle-aged adults without hypertension

Pathophysiology

Normal blood pressure homeostasis is dependent on the equilibration of cardiac output and systemic vascular resistance. However, multiple factors and compensatory mechanisms can lead to dysregulation; insulin resistance, salt intake, obesity, sympathetic nerve stimulation, endothelial dysfunction, and the renin-angiotensin system (RAAS) can lead to hypertension and exacerbate various morbidities [23]. Low salt or low kidney perfusion sensed in the efferent arteriole of the kidney glomerulus are key stimulators of the RAAS, which activates the conversion of renin into angiotensin 1, and quickly into active angiotensin 2 by angiotensin-converting enzyme 1, (ACE-1) leading to the increase in blood pressure [23]. Angiotensin 2 acts at the proximal convoluted tubule in the kidney via the Na-H pumps, increasing sodium retention. In the brain, angiotensin 2 performs three main tasks: it binds the hypothalamus, upregulates thirst, and stimulates water intake [80]. Next, antidiuretic hormone (ADH) is released from the posterior pituitary, increasing the aquaporins in the kidney’s collecting duct [80]. Lastly, baroreflexes become desensitized, diminishing their physiological response to increased vascular pressure [80]. Dysregulation of the RAAS can cause severe consequences; chronic vasoconstriction can induce permanent atrial vessel thickening, hypokalemia related to RAAS overexpression, and endothelial damage by the downregulation of ACE2 [23-25].

ACE2's main role is to reverse the actions of the RAAS, inducing vasodilatation by converting angiotensin-2 into angiotensin-3 while promoting anti-fibrosis and anti-inflammatory properties [81]. It is expressed throughout several organs including the kidney, heart, lungs, and intestines, as well as endothelial cells [82]. The ACE-2 pathway works by utilizing the Mas receptor (MasR) or angiotensin 2 receptor (AT2R) axes, which decreases proliferation and cardiovascular remodeling while increasing vasodilation and nitric oxide production. However, comorbidities, sex, and aging have a negative effect on the expression of ACE-2, leading to a deficiency [24,81]. COVID-19 has a high affinity to ACE2, utilizing it to enter host cells. The arguments for upregulating versus downregulating ACE2 have been ongoing regarding whether it would have a positive or negative effect on patient outcomes and the severity of COVID-19 infections. In previous months, it was recommended by some professionals to avoid non-steroidal anti-inflammatory drugs (NSAIDs) in the treatment regimen as they could increase the expression of ACE2, increasing the entrance of SARS-COV2 into host cells. Newer cases have advocated for increasing the amount of AT2Rs as the viral fusion into the host cell decreases the expression of ACE2, which has increased complications related to pulmonary inflammation, heart failure, hypertension, and increased coagulation [24].

The exact relationships between COVID-19, endothelial damage, and hypertension are not completely understood. The pathophysiology related to hypertension is connected to the production of cytokines, such as TNF-a, IL-6, and IL-17 are believed to stimulate vasoconstriction, ROS production, and sodium reabsorption via the kidney, exacerbating the hypertensive process [83]. Activation of the innate immune system’s complement pathway could adversely affect local and systemic vascular inflammation, potentially leading to hypertensive-related vascular endothelial dysfunction [84]. In one post-mortem study where 71% of subjects had been diagnosed with hypertension, the authors strongly suggested the important role of virus-induced vascular dysfunction [85]. Most cases discovered hypertensive associated signs, such as intimal fibrosis of arteries, arteriolosclerosis, and vascular scarring with virus-like particles found within the endothelium [85]. Indeed, SARS-CoV-2 infection may exacerbate existing conditions of hypertension; perhaps predisposing the patient to more severe infection and complications.

Complications

There is growing evidence to suggest hypertension could account for the severity of COVID-19 pathology; the prevalence of hypertensive patients infected with COVID-19 accounted for 28.8% of the ICU cases, while only accounting for 14.1% of non-ICU cases [14]. In a cohort of 207,079 COVID-19 positive individuals in Argentina, the association of hypertension to the severity of illness was evident. Among all individuals (including non-hospitalized), 19.2% had a history of diagnosed hypertension. This proportion increased to 33.9% in hospitalized patients and 48.9% in ICU patients. In addition, 54.2% of deceased patients had comorbidity of hypertension [72]. A New York study found that hypertensive patients had a mortality rate of 26.7% compared to 21% overall in the cohort of hospitalized patients [70].

One meta-analysis found that hypertension was significantly correlated to COVID-19 mortality, disease progression, ICU care, and ARDS incidence [86]. Furthermore, the same study hypothesized that hypertension caused increased infection rate via upregulation of ACE2 and proposed a relationship with patient age. Increased expression of ACE2, which could theoretically increase the likelihood of initial virion entry and infection. However, ACE2 also mediates anti-inflammatory effects via the RAAS pathway, which would be protective against severe systemic hyperinflammatory complications. Thus, the study hypothesized that older patients, with lowered ACE2 expression, would be less likely to be infected initially, but more likely to develop severe complications, in contrast to younger patients. However, further research would be needed to support this theory [86].

Medications

With no definitive treatments, the management symptoms and adverse complications related to current medication regimens are of the utmost importance. Hypertensive medications have been a major concern; it has been unknown if their use has caused an increase in the infection rate. Reynolds et al. provided that the five main classes of hypertensive drugs, ACE inhibitors, angiotensin-receptor blockers, beta-blockers, calcium-channel blockers, or thiazide diuretics, discovered no significant increase in the likelihood of testing positive for COVID-19 [87].

The efficacy and continuation of angiotensin-converting enzyme inhibitors (ACEi) and angiotensin II receptor blockers (ARB) are still unknown. As previously described, hypertensive and cardiovascular-related complications are related to the deficient expression of ACE2 inhibitory effects of the RAAS during hypertensive states [24]. It is theorized this lack of ACE2 receptors could prevent the initial infection of COVID-19 but once infected, it could lead to a prevention of the anti-inflammatory and lead to a hyper-coagulable state [24]. Interestingly, some research indicates that ACEi and ARB use have no significant correlation with ACE2 expression or initial rate of infection [87,88]. Alternatively, other early studies even found an increased mortality rate in hypertensive COVID-19 patients concurrently taking ACEi or ARB medications, compared to hypertensive patients not taking these medications [70]. The increased severity and mortality were hypothesized to be associated with increased ACE2 expression [70]. However, it should be noted that this study was not intended to examine ACEi/ARB use. Overall, the use of ACEi and ARBs appears to reduce the risk of hypertensive-related COVID-19 complications, thus, decreasing morbidity and mortality [88]. ACE inhibitors appear to stimulate cell-intrinsic antiviral signaling via interferon regulatory factor 3 (IRF3) and demonstrate viral clearance dynamics comparable to normotensive patients. Additionally, ACE inhibitors were associated with reduced leukocyte-endothelium interaction and decreased neutrophil tissue infiltration, which likely prevents lymphocytic lung infiltration, hyperinflammatory states, and lung tissue damage [88]. ARBs did not exhibit these properties and were in some cases associated with a higher incidence of hyperinflammatory states by inducing pro-inflammatory cytokines CCL3 and CCL4 [88]. The negative effects of ARBs remain unclear as ARB treatment has also demonstrated protective effects in COVID-19 infections. For instance, one study did show evidence of losartan, an ARB, improving respiratory function [89]. Another study emphasized high cases of hypokalemia (62%) in hospitalized individuals due to high expression of the RAAS and suggested ARB for treatment [25]. The relationship between ACEi and ARB treatment to pathophysiology is unclear at best, yet the consensus remains to continue current ACEi and ARB regimens for hypertensive patients to prevent COVID-19 complications.

No studies could be found testing to potential benefits of beta-blocker therapy and COVID-19 infection. It is believed it could be beneficial in patients as its mechanism of action decreases the RAAS upregulation, decreasing preexisting hypertension, as well as decreasing ACE2 and thus cellular entry [90]. Extreme caution must be taken, as low ACE2 hyper-coagulation described earlier is still a potentially serious adverse event [24].

Novel drug therapies should consider mimicking ACE2 receptors, which cannot adhere to host membranes. Such therapies are likely to bind viral loads in the plasma while preventing the adherence to host cells [81]. Human recombinant soluble ACE2 has already been previously approved for the treatment of ARDS and would make it much faster to transfer into clinical trials [81]. Another novel therapy to consider is alamandine, a heptapeptide apart of the noncanonical pathway that is catalytically activated by ACE2 and mimics the effects of Ang-(1-7), preventing fibrosis and stimulating vasodilatation [91]. Other antihypertensive agents, such as dihydropyridine calcium channel blockers (CCB) have been shown to increase ACE2 Ang(1-7) [81]; moreover, nifedipine and amlodipine CCB has been shown to decrease mortality and intubation in the elderly [92].

Other approaches look to prevent further exacerbation of damage to vascular and endothelial tissue contributing to hypertension. Some approaches are looking at altering the immune complement system concerning endothelial dysfunction. Monoclonal antibody therapies, such as eculizumab work against the C5 complement protein and have exhibited real potential in MERS-CoV and SARS-CoV animal models [85].

Management

The COVID-19 hospitalization rate in the United States represents only a minority of individuals (4.6 per 100,000). However, concerns regarding vulnerable communities should be on high alert. Of all the hospitalized cases in the United States, 91% of those reported at least one underlying medical condition, one of the most common being hypertension [93]. Withholding COVID-19 care can exacerbate or create an increasing scale of comorbidities including cardiac disease, renal failure, and hypertension [81,94]. While uncertainties underlay the significant uncertainty on the ACE1/ACE2 receptor ratio, there has been no increase in the ACE2 receptor with the use of ACEi and no measurable increased risk with ARB [81]. There are no clinically significant findings that any RAAS-inhibiting drugs should be discontinued or limited in any way but further longitudinal studies with a comprehensive analysis should be considered in future studies, as they are effective in the treatment of hypokalemia in hypertensive COVID-19 patients [89,94]. Exacerbation prevention of hypertension, as well as cell infection, should be further considered and tested with the use of human recombinant ACE2 soluble drugs [81]. These could prove as effective treatment options in the future of COVID-19 care. Factors should also look to lifestyle modification continuation, including maintaining a low salt and well-balanced diet, exercise, weight loss focus, and avoiding high protein diets depending on the underlying cause of hypertension.

COVID 19 and diabetes

Incidence and Prevalence

Many articles and online sources have been provided to track the number of cases of COVID-19 [95-97], and some have more specifically focused on the prevalence of the virus among patients with diabetes [20,21,98-100]. While this has been a popular area of focus, studies have shown conflicting data. A study done in China reported the prevalence of diabetes with COVID-19 to be 8.2% [101]. One report that examined six different studies conducted in China has shown the prevalence of patients with diabetes and COVID-19 to be, on average, about 10.6%[99]. The CDC reports a similar statistic at 10.9% [100]. Despite these similar figures, an Italian study reported that nearly 36% of patients with COVID-19 also have diabetes [21]. This figure has been reported to be as high as 58% in a small study in the United States [20]. These wide variations in the prevalence of diabetes as an underlying condition may be linked to different sampling methods and heterogeneity of different cohorts. Since some studies may have used a cohort consisting of proportionally more ICU (severe cases) COVID-19 patients, Table 3 separates the prevalence among ICU and non-ICU patients. The idea of separating ICU patients and non-ICU patients (in Table 3) is to minimize the heterogeneity of the cohorts and to clarify separate correlations between diabetes and ICU vs non-ICU incidence. The separation may also highlight if elevated diabetes incidence is correlated with increased severity of COVID-19 (resulting in ICU care).

**Table 3:** Prevalence and severity of patients with diabetes in COVID-19 across various studies and countries NR: no response

Studies by country	Sample Size (N)	Diabetes Mellitus (N, %)	General Findings	
			Non-ICU Care (%)	ICU Care (%)
China				
Liu et al. [102]	61	5 (8.2%)	4.5%	17.6%
Wu et al. [31]	201	22 (10.9%)	5.1%	19.0%
Zhang et al. [103]	140	17 (12.1%)	11.0%	13.8%
Huang et al. [104]	41	8 (15%)	8.0%	25.0%
Guan et al. [101]	1590	130 (8.2%)	NR	14.6%
Italy				
Onder et al. [21]	355	126 (35.5%)	NR	NR
USA				
Bhatraju et al. [20]	24	14 (58.0%)	NR	NR

Pathophysiology

The pathophysiology of both type 1 and type 2 diabetes have been studied previously and are well-understood. However, a deeper look into how the pathogenic mechanism of diabetes affects that of COVID-19 continues to be examined. It is known that diabetes is a chronic inflammatory condition that can affect the body’s response to pathogens. Hyperglycemia and insulin resistance are shown to increase the synthesis of advanced glycation end products (AGE), pro-inflammatory cytokines, as well as adhesion molecules that promote tissue inflammation [27]. Diabetes also inhibits the actions of neutrophils, limiting chemotaxis, phagocytosis, and intracellular killing of pathogens [28]. In addition, diabetes can delay the initial activation of helper T Cells, often presenting with late inflammatory responses [28]. The inflammatory process and impaired adaptive immunity serve as the underlying mechanism behind the increased predisposition to infections and dysfunctional inflammatory response.

The potential mechanisms behind the increased susceptibility to COVID-19 among patients with diabetes have also been examined with ACE2 [24,26,89,105]. One study in rodent models of diabetes has shown there is altered expression of ACE2 in alveolar type 2 cells, myocardium, kidney, and pancreas, which favors increased affinity for SARS-CoV-2 [26]. These rodent models with diabetes have also shown to have increased expression of ACE2 in the lung, kidney, heart, and pancreas [26]. These findings support another study conducted to examine the effects of diabetes in human-mouse models infected with MERS-CoV [105]. It was shown that the disease was more severe and prolonged in the mice with diabetes, who also presented with abnormal CD4+ T cell counts as well as elevated IL17a [105]. Currently, human patients with diabetes have shown consistent laboratory figures with low CD4+ and CD8+ T cell counts as well as elevated Th17 cells and other pro-inflammatory cytokines [105].

While these reports have suggested the effects of COVID-19 on patients with diabetes in general, some studies have focused on examining the relationship and contrast between COVID-19 and type 1 diabetes from type 2 diabetes [78,106]. One report has indicated an increase in the prevalence of patients with type 1 diabetes with COVID-19, potentially due to uncontrolled diabetic ketoacidosis and delayed hospital admission [78]. Because of this delay, it is even more necessary for patients with Type 1 diabetes and elevated HbA1c levels to closely monitor the risk of developing diabetic ketoacidosis [78]. This would include re-educating patients about typical symptoms, how to accurately measure urine and blood ketones from home, and when to seek professional medical advice to lower the risk of diabetic ketoacidosis complications [78].

One case study has suggested the effects COVID-19 may have on precipitating diabetic ketoacidosis in newly diagnosed patients with type 1 diabetes [106]. One previously healthy patient who tested positive for SARS-CoV-2 presented laboratory values with hyperglycemia, high anion gap metabolic acidosis, and ketonemia, confirming the diagnosis of diabetic ketoacidosis [106]. As previously stated, the expression of ACE2 in the lungs and pancreas has served as an entry point for SARS-CoV-2, then its expression is downregulated after the virus has been endocytosed [24,26,89,105]. Entry of SARS-CoV-2 may directly aggravate and damage the pancreatic islet cells that produce insulin; downregulation of ACE2 can also cause unopposed angiotensin II expression, impeding insulin secretion [106]. These two potential mechanisms can worsen pancreatic beta-cell function in those with existing type 1 diabetes and lead to the precipitation of diabetic ketoacidosis [106].

Complications

Diabetes mellitus appears to have a clear correlation with increased severity of COVID-19 and increased ICU placement. Based on numerous studies (such as those presented in Table 3), diabetes mellitus tends to have a prevalence of around 5% in non-ICU patients (higher in some cohorts), whereas the prevalence ranges between approximately 15-30% in ICU patients (in most cohorts) [12,20,21,31,68,100-104,107]. One factor to induce severe COVID-19 may be diabetes and hyperglycemia exacerbating inflammatory responses by upregulation of TNF-a and IL-10 [108,109]. One retrospective study linked diabetes mellitus to a significantly increased risk of mechanical ventilation and mortality. The same severe COVID-19 patients with diabetes were observed to have increased neutrophil and leukocyte counts, but decreased lymphocyte counts, with their non-diabetic counterparts. Additionally, severe COVID-19 with diabetes was linked to the elevation of inflammatory markers IL-6, IL-8, hsCRP, procalcitonin, and IL-2 receptors [107]. The findings may suggest diabetes mellitus could predispose COVID-19 patients to more severe infection and indicate possible secondary bacterial infections. Numerous studies have also linked diabetes (and obesity) to dysregulation of the inflammatory response by upregulation of Th1 and Th2 cells and induced dysfunction of Th17 and Treg cells [108, 110-112]. The imbalance between proinflammatory and anti-inflammatory expression may be an important factor in linking diabetes to increased severity and complications of COVID-19. In addition, the hypoglycemia and subsequent glycosylation of IgE Fc domains may also induce dysfunction of the adaptive immune response and increase the risk of complications and mortality [107].

Medications

While there are many life-changing antidiabetic medications, some studies have reported these same medications to have varying effects on COVID-19 patients. Studies have shown a relationship between the involvement of ACE2 with that of COVID-19 infection and the pathogenic mechanism of diabetes [24,26,89,105]. Although insulin administration has shown to attenuate the expression of ACE2, other common antidiabetic medications have shown opposite effects [26]. One study has reported that hypoglycemic agents including glucagon-like peptide-1 (GLP-1) agonists, such as liraglutide, and thiazolidinediones (TZDs) such as pioglitazone, have shown to upregulate the expression of ACE2 [26].

Another study looked at patients with COVID-19 and diabetes comparing the laboratory values between those who took insulin supplementation in comparison to those who did not [113]. The results indicated that, in comparison to non-insulin users, patients who used insulin as a part of their glucose-lowering medication regimen showed lower levels of albumin, high C-reactive protein (CRP), and procalcitonin, erythrocyte sedimentation rate, as well as higher blood glucose and HbA1c [113]. Despite these differences, patients with severe COVID-19 illnesses showed no significant variances in the percentages between patients taking insulin versus those who do not [113]. With regards to specific antidiabetic medications, patients using the biguanide metformin have reported laboratory values with increased albumin, but lower levels of urea and IL6 [113]. For those using secretagogues, such as sulfonylureas or meglitinides, patients exhibited significantly lower white blood cell count, fewer neutrophils, lower creatine kinase, CRP, and IL6 compared to those who do not [113]. Similar laboratory values were seen in patients using a-glycosidase or DPP-4 inhibitors in comparison to those who do not [113]. While a multivariable regression was performed in this study depicting a greater risk of poor prognosis among insulin users in comparison to non-insulin users, none of the glucose-lowering medications mentioned earlier were associated with an increased risk of hospital death [113].

Management

While a majority of COVID-19 infected individuals may have minor symptoms and will not require hospitalization, patients with an immunocompromised status, such as those with diabetes, can present with more serious complications. Since patients with diabetes often have a strict medication regimen, this is especially important to maintain if they also have COVID-19. This includes the need to frequently monitor glucose levels, have a healthy diet, hydrate adequately, and adjust their glucose-lowering medication with the guidance of healthcare providers [113]. The use of NSAIDs, such as ibuprofen, may typically aid in symptom relief; however, current research suggests that NSAIDs increase ACE2 expression, which has shown to increase in affinity for SARS-Cov-2 [26,28,89]. This suggests that acetaminophen, rather than ibuprofen, be the antipyretic drug of choice.

With regards to patients with diabetes, there is an emphasis on proper glycemic control, especially if infected with COVID-19 [114]. Proper management of patients with diabetes depends on the type of antidiabetic medication the patient is currently using. For severely ill patients taking metformin, it is advised to temporarily stop due to the risk of lactic acidosis and hypoxia [114]. Sodium-glucose Cotransporter-2 (SGLT2) inhibitors present with the risk of dehydration and euglycemic ketoacidosis, which should also be temporarily stopped in severely ill patients [114]. GLP-1 receptor agonists should also be stopped in severely ill patients to prevent the risk of aspiration [114]. DPP-4 inhibitors, however, have a low risk of hypoglycemia and, therefore, can continue to be used in non-critically ill patients [114]. Although insulin requires frequent monitoring to maintain proper glucose levels, it remains to be the drug of choice in patients with diabetes and COVID-19 [114].

Diabetogenic Effect of COVID-19

As previously mentioned, the SARS-CoV-2 infection of pancreatic cells, via ACE2 receptors, may influence insulin production, and thereby precipitate diabetogenic effects [106]. A similar relationship was observed in SARS-CoV-1 infections, as SARS-induced pneumonia was correlated to higher incidences of acute-onset diabetes, compared to non-SARS pneumonia cases [115]. Similar to the COVID-19 diabetogenic hypothesis, acute-onset diabetes observed in SARS was believed to occur via residual islet damage following SARS-CoV-1 entry of pancreatic cells via ACE2 receptors [116]. Currently, an international team of diabetes researchers is composing a global registry of patients with COVID-19-related acute-onset diabetes, called coviDIAB, to further examine this correlation [115]. Regardless of the exact etiology, there is an indication to suggest that COVID-19-induced hyperglycemia is associated with overall poorer outcomes, higher frequency of complications, and higher mortality rates [116].

COVID-19 and dyslipidemia

Incidence and Prevalence

Dyslipidemia is one of the hallmarks of metabolic syndrome and is characterized by abnormal cholesterol levels or elevated triglycerides [22]. Dyslipidemia can be defined as having triglyceride levels >150 mg/dL. Additionally, decreased levels of high-density lipoprotein (HDL) or “good” cholesterol, can be used as a clinical indicator of metabolic syndrome. This is defined as males having an HDL level <40 mg/dL or females <50 mg/dL. Low-density lipoprotein (LDL) or “bad” cholesterol may be normal or slightly elevated in metabolic syndrome and is not used as a clinical indicator [117].

In 2015-2016, it was found that 18% of adults 20 years or older in the United States had HDL cholesterol levels <40 mg/dL [118]. Using NHANES data from 2007-2014, it was found that 25.9% of American adults 20 years or older, had triglyceride levels >150 mg/dL [119]. Hypertriglyceridemia and low-HDL cholesterol are associated with an increased risk for cardiovascular disease [120-124].

Minimal research has been done to show the connection between hyperlipidemia and the risk of COVID-19. In a study conducted with 200 COVID-19 patients, 46.2% of individuals had a past medical history of hyperlipidemia. However, hyperlipidemia was not shown to be a significant indicator for in-hospital mortality, use of supplemental oxygen, or intubation [125]. In a study conducted with 4510 participants of the UK Biobank (Stockport, Greater Manchester, UK), 1326 individuals were positive for COVID-19. Analysis showed that elevated cholesterol levels before infection were not significantly associated with COVID-19 risk [126]. There is currently no literature found on hypertriglyceridemia and the associated risk of COVID-19.

Pathophysiology

The SARS-CoV-2 attaches to the ACE2 receptor through its spike glycoprotein (“S protein”) [8, 9]. Lipid rafts, which are components of the plasma membrane and are rich in cholesterol, are thought to play an important role in facilitating the attachment of the S protein to the ACE2 receptor; therefore, it is suggested that hypercholesterolemia may facilitate lipid rafts, and enhance viral entry into the cell [34].

It has also been suggested that dyslipidemia may exacerbate a pro-inflammatory state. Based on a meta-analysis of seven cohort studies, it seems that dyslipidemia is associated with an increased incidence of severe COVID-19 cases [127]. The accumulation of LDL may induce inflammasome activation and release of pro-inflammatory cytokines, such as IL-1b and IL-18, and low levels of HDL have been negatively correlated with CRP levels. In COVID-19 cases [128, 129], high levels of pro-inflammatory cytokines have been associated with cytokine storm syndrome and higher incidences of severe outcomes [130].

Complications

In individuals sick with COVID-19, abnormal cholesterol levels can be seen and may serve as an indicator of the severity of the disease. In a study by Wei et al., 597 COVID-19 patients were identified [131]. Based on the clinical severity of their symptoms, they were placed in mild (n=394), severe (n=171), and critical (n=32) categories. Fifty healthy individuals served as the control. It was seen that LDL-c and total cholesterol (TC) levels were lower in COVID-19 patients, as compared to the normal subjects. Additionally, there were significant decreases in LDL-c and TC levels with increasing clinical severity of COVID-19. HDL-c was decreased in critical cases only [131].

Similar results were seen in a study of 75 COVID-19 patients who were classified into mild/moderate (n=26), severe (n=39), and critically severe (n=10) categories, based on the severity of their symptoms. It was found that TC was significantly abnormal in critically severe cases of COVID-19, as compared to those with mild/moderate cases [132].

In a small cohort of 21 COVID patients, 31 healthy controls, and 21 COPD controls, it was seen that LDL and TC decreased throughout the time COVID-19 patients were admitted to the hospital. These levels were restored upon discharge, except HDL, which decreased over the time of admission [133]. Lastly, a study was conducted with 62 COVID-19 patients, classified as non-severe with cardiovascular disease (CVD) (n=16), non-severe without CVD (n=22), severe with CVD (n=17), severe without CVD (n=7). HDL was found to be higher in the severe with CVD group, as compared to the non-severe with CVD group [134].

Hu et al. found similar results in their study of 71 COVID-19 patients, where TC, HDL, and LDL were seen to decrease [123]. Wang et al. found HDL-c to be below the normal range in their study of 228 COVID-19 patients. Furthermore, low HDL-c was associated with an increased risk for severe clinical presentations, after adjusting for age, gender, and underlying medical conditions [135].

Medications

Lipid rafts are thought to enhance the attachment of SARS-CoV-2 S protein to an individual’s ACE2 receptors [34,136]. It has been proposed that statins may disrupt this process, as they reduce levels of cholesterol by inhibiting the HMG-CoA reductase enzyme in cholesterol synthesis, thereby reducing lipid rafts [34,136]. A retrospective cohort study of 13,981 COVID-19 patients in the Hubei province of China supports this correlation between statins and COVID-19 severity. The study found that all-cause mortality in 1,219 COVID-19 patients receiving statin treatment was 5.2% compared to 9.4% in the non-statin patient group [137]. Although the reduction of lipid rafts hypothesis may hold some validity, the observed beneficial effects of statins may also be due to their immunomodulatory effects [34,137]. For instance, statins have been found to lower CRP levels and moderately reduce the risk of pneumonia in healthy adults [136]. Nevertheless, further research is needed to see if statins can be used as therapeutics in the prevention and treatment of COVID-19 [138].

Management

Based on the current body of research, it cannot be determined if having hyperlipidemia before SARS-CoV-2 infection will increase the risk of COVID-19 [125,126]. However, current research suggests that abnormal levels of LDL and TC can be found in individuals sick with COVID-19 [123,131-134]. Furthermore, the clinical severity of the infection may be determined by the decrease in LDL and TC [131,132,134]. Conflicting information has been seen regarding HDL levels and whether decreases are seen, and which acuity of patients [123,131,133-135]. Further research is needed to identify the role of pre-existing hyperlipemia as a risk factor for COVID-19, particularly triglyceride levels, in addition to the use of cholesterol levels as prognosis indicators of COVID-19 severity. Table 4 summarizes patient demographics and corresponding general findings of several studies of COVID-19 patients in relation to dyslipidemia.

**Table 4:** Summary of patient demographics and corresponding general findings in eight meta-analysis cohort studies of COVID-19 patients in relation to dyslipidemia COVID-19: coronavirus disease 2019; LDL: low-density lipoprotein; HDL: high-density lipoprotein; TC: total cholesterol

Study	Participant Demographics	General Findings
Wei et al., 2020 [131]	597 COVID-19 patients (mild: 394, severe: 171, critical: 32) Normal subjects n=50	LDL-c and TC levels lower in COVID-19 pts compared to normal subjects. Significant difference/decrease of LDL-c and TC with increasing severity of disease. HDL-c was decreased in critical cases only.
Fan at al., 2020 [133]	21 COVID-19 patients (healthy: 31, COPD: 21)	LDL and TC decreased throughout time pts admitted. Returned to higher levels at discharge. HDL decreased over time of admission and was not restored at time of discharge.
Zhao et al., 2020 [132]	75 COVID-19 patients (mild/moderate: 26, severe: 39, critically severe: 10)	TC was significantly abnormal in critically severe cases compared to moderate patients.
Raisi-Estabragh et al., 2020 [126]	4510 UK Biobank participants (COVID-19 positive=1326)	High cholesterol not significantly associated with COVID-19 risk
Xie et al., 2020 [134]	62 COVID-19 patients (non-severe: 38 (with CVD: 16; without CVD: 22); severe: 24 (with CVD: 17; without CVD: 7).	HDL higher in the severe COVID (with CVD) group compared to the non-severe (with CVD)
Palaiodimos et al., 2020 [125]	200 COVID-19 patients	46.2% of patients had hyperlipidemia. Hyperlipidemia is not significant for in-hospital mortality, needing O2, or needing intubation.
Hu et al., 2020 [123]	71 COVID-19 patients; 80 age-matched healthy controls	Decreased TC, HDL, and LDL cholesterol in COVID-19 patients. TC, HDL, and LDL returned to higher levels upon discharge in a single patient followed.
Wang et al., 2020 [135]	228 COVID-19 patients	HDL-c below normal range in COVID patients. Those with lower HDL were at higher risk of developing severe events even after adjusting for age, gender, and underlying diseases

## Conclusions

The goal of this study was to examine the relationship between metabolic syndrome and pathophysiology, incidence, prevalence, and severity of COVID-19. The management and medications of metabolic syndrome were also reviewed for their influence on COVID-19 pathophysiology. Obesity was generally considered the second most prevalent comorbidity in severe COVID-19 patients. It was correlated to higher morbidity and mortality, likely due to factors of decreased chest wall compliance and chronic inflammation. Furthermore, factors such as increased ACE2 expression and decreased miRNA-146 were proposed as causes of increased infection rate and morbidity respectively, although further research was needed. Management strategies of behavioral lifestyle modifications and bariatric surgery (severe cases) were correlated with improved outcomes of COVID-19. No clear conclusions could be made on the legitimacy of the obesity paradox. Hypertension was the most frequent comorbidity in severe cases of COVID-19 and was linked to elevated rates of mechanical intubation, ICU care, ARDS, and mortality. The pathophysiology was proposed to be caused by a RAAS dysregulation secondary to occupancy of ACE2 (which normally exerts an inhibitory effect on the RAAS axis) by SARS-CoV-2 infection. Theoretically, the existent RAAS dysregulation in hypertension may be exacerbated by SARS-CoV-2 interaction with ACE2. Current research on ACEi and ARB medications was highly conflicting, but consensus remains to continue the current regimen in hypertensive COVID-19 patients. The potential benefits of recombinant medications were also discussed.

Diabetes mellitus was concluded to have a significant correlation with increased rates of ICU hospitalization and mortality in COVID-19 patients. Diabetic patients are at higher risk of having COVID-19 infection and severe outcomes. It was theorized that chronic immune dysregulation and potential changes in ACE2 expression in diabetes mellitus were the main factors in exacerbating COVID-19 illnesses. Diabetic medications were believed to alter ACE2 expression but showed no significant change in mortality risk. Dyslipidemia had no significant correlation with COVID-19 incidence, prevalence, morbidity, or mortality. No clear conclusions could be made due to a lack of extensive research. However, it was found that statin treatment regimens may have a protective role against COVID-19 complications, through lipid rafts and immunomodulatory mechanisms. Aside from dyslipidemia, the metabolic syndrome comorbidities demonstrated a significantly increased risk of morbidities/complications and mortality in COVID-19 patients. The pathophysiology was most often related to immune dysregulation or alterations in ACE2 expression.

Future research should examine the ACE2-related pathophysiological mechanisms, as these remain unclear yet specific to COVID-19 and high-risk comorbidities of hypertension, obesity, and diabetes. ACE2 regulation may be a specific and effective potential target for future COVID-19 treatments.
